# Molecular Analysis of the Avian H7 Influenza Viruses Circulating in South Korea during 2018–2019: Evolutionary Significance and Associated Zoonotic Threats

**DOI:** 10.3390/v13112260

**Published:** 2021-11-11

**Authors:** Bao Tuan Duong, Jyotiranjan Bal, Haan Woo Sung, Seon-Ju Yeo, Hyun Park

**Affiliations:** 1 Zoonosis Research Center, Department of Infection Biology, School of Medicine, Wonkwang University, Iksan 54538, Korea; bao2dt@gmail.com (B.T.D.); jyoti_micro@yahoo.co.in (J.B.); 2 College of Veterinary Medicine, Kangwon National University, Chuncheon-si 24341, Korea; 3 Department of Tropical Medicine and Parasitology, Seoul National University College of Medicine, Seoul 03080, Korea

**Keywords:** H7, avian influenza, highly pathogenic

## Abstract

Avian influenza virus (AIV) subtypes H5 and H7, possessing the ability to mutate spontaneously from low pathogenic (LP) to highly pathogenic (HP) variants, are major concerns for enormous socio-economic losses in the poultry industry, as well as for fatal human infections. Through antigenic drift and shift, genetic reassortments of the genotypes pose serious threats of increased virulence and pathogenicity leading to potential pandemics. In this study, we isolated the H7-subtype AIVs circulating in the Republic of Korea during 2018–2019, and perform detailed molecular analysis to study their circulation, evolution, and possible emergence as a zoonotic threat. Phylogenetic and nucleotide sequence analyses of these isolates revealed their distribution into two distinct clusters, with the HA gene sharing the highest nucleotide identity with either the A/common teal/Shanghai/CM1216/2017, isolated from wild birds in Shanghai, China, or the A/duck/Shimane/2014, isolated from Japan. Mutations were found in HA (S138A (H3 numbering)), M1 (N30D and T215A), NS1 (P42S), PB2 (L89V), and PA (H266R and F277S) proteins—the mutations had previously been reported to be related to mammalian adaptation and changes in the virulence of AIVs. Taken together, the results firmly put forth the demand for routine surveillance of AIVs in wild birds to prevent possible pandemics arising from reassortant AIVs.

## 1. Introduction

Influenza A viruses (IAV), belonging to the family *Orthomyxoviridae,* possess the ability to adapt to multiple hosts, including wildlife, domestic animals, and humans, causing yearly outbreaks with high rates of morbidity and fatality [1]. The virus can reassort or mutate continuously to acquire point mutations while circulating in several hosts, ranging from aquatic birds to mammals, including humans. IAV can be transmitted from wild birds to domestic birds, causing large scale mortality, sporadically followed by transmission to humans, commonly referred to as zoonotic infections [2]. Genetic reassortments between avian influenza viruses affecting different species, and their attainable human transmission, continue to pose a serious challenge to the prediction of, and ground level readiness to deal with, the emergence of new pandemic viruses.

Among the IAVs, H5 and H7 subtypes have been reported to undergo genetic mutations in their HA gene, and thereby transform from low pathogenic (LP) to high pathogenic (HP) [3,4]. Wild birds are well known as natural factors for IAV re-assortment, since they assist in spreading the virus to geographically distinct regions across major water bodies, through migration [5]. An interesting characteristic of the AIV H7 subtype is interspecies transmission, which determines its evolution and ecology [6]. The H7-subtype HA gene has been found in combination with nine NA subtype genes [7]. To date, H7N2, H7N3, H7N4, H7N7, and H7N9 subtypes have been reported to infect humans [7,8,9], H7N9 being the most virulent. The H7N7 AIV originated in 1902 in Italy as a virus infecting chickens [10], followed by the first reported human infection in Netherlands [8] in 2003. H7N7 AIV strains were further detected in migratory birds in China in 2013 [11]. Recently, a new strain of H7N7 AIV, designated as CM1216, was isolated from wild birds in Shanghai, which is an important stopover place for many migratory birds in the East Asia-Australia migratory flyway, suggesting possible genetic exchanges between the various H7 isolates [12]. Four genetically distinct strains of H7 LPs had been detected in domestic duck farms in the Republic of Korea during 2008–2011, which were closely related to viruses circulating in migratory birds [13]. H7-subtype LP isolates from wild bird habitats in the Republic of Korea have been characterized since 2010 up to early 2017 [14].

The Republic of Korea has a climate with long and dry winters, which seem to provide preferrable conditions for the continued evolution of influenza viruses by increasing the possibility of mixed genomes occurring between lineages within subtypes [15]. Therefore, surveillance and effective monitoring of AIVs has immense practical significance in the prevention of possible pandemics.

In this study, through national surveillance of IAVs, we isolated nine strains of H7N7 from wild birds during 2018, and four strains of H7N7 and one strain of H7N3 during 2019 in the Republic of Korea. To gain extensive knowledge about the evolution pattern of these viruses and the possible zoonotic threats thereby, we performed phylogenetic analysis based on each segment of the isolates, and compared them with other potent IAV sequences. In addition, we investigated the possible mutations affecting crossover from one species to another, and assessed the possible evolution of H7 IAV subtypes and their potential as a future zoonotic threat.

## 2. Materials and Methods

### 2.1. Sampling and Virus Isolation

During December 2018 and 2019, fresh fecal samples were collected from wild bird habitats early in the morning before sunrise. Among the 2019 isolates, KNU18-104 (A/wild duck/South Korea/KNU18-104/2018(H7N7)) and KNU18-113 (A/wild duck/South Korea/KNU2018-113/2018(H7N1)) samples were isolated from the feces of wild ducks, obtained nearby Geumho river, Gyeongsang province (35°54′34.31″, 128°49′43.61″) and Wonju river, Wonju-si, Gangwon-do (37°22′56.11″, 127°56′29.93″), respectively. KNU18-119 (A/White-fronted Goose/South Korea/KNU18-119/2018(H7N7)) samples were isolated from the feces of white fronted geese, obtained nearby Sihwa lake, Hwaseong-si, Gyeongii-do (37°13′49.20″, 126°40′16.65″). KNU18-106~110 (A/wild duck/South Korea/2018(H7N7)) were isolated from the feces of wild ducks, obtained nearby Namdae stream, Yangyang-gun, Gangwon province. Among the 2019 isolates, KNU2019-14 (A/Mallard (Anas platyrhynchos)/South Korea/KNU2019-14/2019(H7N7)) was isolated from the feces of mallards, obtained nearby Gunpyeong-ri, Osong-eup, Chungbuk province ((36°37′31.36″, 127°20′59.71″), KNU2019-25 (A/Mallard/South Korea/KNU2019-25/2019(H7N3)) from Sangrim-ri, Jillyang-eup, Gyeongsangbuk province (36°45′27.47″, 127°29′8.11″), KNU2019-30 (A/Mallard/South Korea/KNU2019-30/2019(H7N7)) from Sin-dong, Chuncheon, Gangwon province (37°55′10.37″, 127°44′10.07″), KNU2019-33 (A/Mallard/South Korea/KNU2019-33/2019(H7N7)) from Gahyeon-dong, Weonju, Gangwon province (37°23′0.40″, 127°56′28.28″), and KNU2019-39 (A/White fronted Goose/South Korea/KNU2019-39/2019(H7N7)) from the feces of mallards obtained nearby Songchon-dong, Paju-si, Gyeonggi province (37°45′17.28″, 126°42′1.16″). The fecal samples were transported in viral transport medium (VTM) supplemented with antibiotics. Two hundred microliters of VTM containing the fecal samples were inoculated into the allantoic cavities of 9–11-day-old specific-pathogen-free (SPF) embryonated eggs. The eggs were then incubated at 37 °C, and embryonic status was checked every day thereafter. After 5 days of incubation, the eggs were chilled, and allantoic fluids were subsequently harvested and tested for the presence of viruses by estimating hemagglutination activity using chicken erythrocytes, or by genetic confirmation through qRT-PCR using gene-specific primers. For host species determination, the mitochondrial gene and cytochrome c oxidase I (COI) gene were amplified with specific primers.

### 2.2. RNA Extraction and Next-Generation Sequencing (NGS)

A NucleoSpin RNA kit (MACHEREY-NAGEL, Düren, Germany) was used to extract viral RNA directly from the allantoic fluid of embryonated chicken eggs (ECEs) according to the manufacturer’s instructions. In a nutshell, virus-containing egg fluid was combined with β-mercaptoethanol-containing lysis buffer. Filtration through a NucleoSpin filter lowered the suspension’s viscosity, and ethanol was added to alter the RNA-binding conditions. Desalting was accomplished by adding a membrane desalting solution to solubilized RNA attached to a NucleoSpin RNA column membrane. Because rDNase treatment could potentially reduce RNA stability, the DNA digestion step was skipped. After washing the desalted membrane with wash buffers, elution was conducted immediately. The first-strand cDNA was transcribed using a Superscript III first-strand cDNA synthesis kit (Invitrogen, Carlsbad, CA, USA) in a final volume of 20 µL, according to the manufacturer’s instructions, using a universal primer for influenza A virus (Uni12: 5′-AGCRAAAGCAGG-3′).

NGS was conducted by GnCBio (Daejeon, Korea) following the HiseqX method, as reported previously [16]. The cDNA library was constructed using fragments amplified by the REPLI-g SensiPhi DNA polymerase enzyme in the QIAGEN (Hilden, Germany) QIAseq FX single-cell RNA library kit. Qualitative evaluation of the constructed library was performed using the LightCycler qPCR instrument (Roche, Penzberg, Upper Bavaria, Germany), and its size was verified using Agilent TapeStation D5000 ScreenTape system (Agilent, Santa Clara, CA, USA). The library was placed into a flow cell for cluster synthesis, where fragments were immobilized on a lawn of surface-bound oligos that were complementary to the library adapters. Through bridge amplification, each fragment was amplified into different clonal clusters. After cluster creation, the templates were ready to be sequenced. For analysis, the sequencing data was converted to raw data. Non-influenza virus reads were eliminated using Deconseq (iden = 60), and raw sequence reads were quality-trimmed using “trim galore” (q = 20). The amount of data was adjusted using a Python script up to 600,000 reads. Using Gsmapper (iden = 80, mL = 40), a database of only segments four (HA), six (NA), and eight (NS1) from the influenza virus genome information in NCBI was created and aligned to those of the reference. Using the obtained consensus, the open reading frame (ORF) was observed, and a result with an ORF similar to the reference was adopted.

### 2.3. Phylogenetic Analysis

In this study, the gene sequences of influenza A virus, available from the GISAID (https://platform.gisaid.org/epi3/frontend#22c194) and NCBI, were downloaded on 31 May 2021. Each nucleotide sequence of the isolated subtype virus was queried against the downloaded database using nucleotide BLAST (NCBI) with default parameters, and sequences of the top 100 hits were collected. Thus, for each gene, 800 similar sequences were obtained, and sequences with the same strain name were removed. Phylogenetic analysis was performed for two rounds using MUSCLE and the maximum-likelihood method implemented in MEGA v.6.017. In the first round, 200 bootstrap replicates were run for all sequences. Based on the result of first round, in the second round, we selected several representative reference sequences, and pulsed H7N7 Korea strain sequences, forming smaller data sets. Phylogenetic analysis was repeated using the method described above with bootstrap 1000.

### 2.4. Temporal Dynamics of the H7 Isolates

To understand the evolutionary dynamics of seasonal influenza in the Republic of Korea from 2018 to 2019, all available HA sequences of the H7 isolates during this period were compared with those of other AIVs. For the H7 hemagglutinin genes of the isolates from Korea, a Bayesian time-resolved phylogenetic tree was created using BEAST v1.10.4. We employed the SRD06 nucleotide substitution model with a log-normal distributed uncorrelated relaxed clock. For the analysis, the Bayesian skyline coalescent was chosen as tree prior. Tracer v1.7.1 used a Markov chain Monte Carlo (MCMC) approach, sampling every 5000 steps to obtain a post burn-in effective sample size (ESS) of at least 200 in all parameters. The maximum clade credibility tree was obtained by using Tree Annotator v1.10.4 with 10% burn-in. The final MCC tree was visualized using FigTree v1.4.4.

### 2.5. Accession Numbers

Nucleotide sequences were deposited in the GenBank with the accession numbers mentioned in Table S1.

## 3. Results

### 3.1. Isolation and Genomic Characterization of H7 IAVs during 2018–2019 in the Republic of Korea

During 2018–2019, we isolated a total of 14 H7 isolates from wild bird habitats in the Republic of Korea, out of which, nine were isolated in 2018, and five in 2019. Genome sequence information of the isolates was deposited in GenBank with the accession numbers mentioned above. GenBank accession numbers of the eight gene segments and the highest nucleotide identities from the GenBank database are shown in Tables S1–S13, with sequence identities from 98.16 to 99.12% when compared to other IAV sequences.

In the isolate KNU2019-14 from 2019, surface gene HA and polymerase basic protein (PB)1 were closely related to A/common teal/Shanghai/CM1216/2017 (H7N7), which originated from China, whereas NA was closely related to A/mallard duck/Georgia/10/2016 (H7N7), with nucleotide identities of 99.51%, 99.64%, and 97.88%, respectively—the HA, PB1, and NA segments of another isolate KNU2019-33 were closely related to A/common teal/Shanghai/CM1216/2017 (H7N7). Most of the H7N7 isolates had homology associated with the isolates from China, except for one H7N3 isolate KNU2019-25, where HA was closely related to A/Jiangsu/1/2018 (H7N4). However, PB1 and NP were closely associated with sequences from the isolates obtained from ducks in Cambodia, namely H10N7 and H7N4, respectively.

### 3.2. Molecular Characteristics of the H7 Isolates of 2018–2019 from the Republic of Korea

The 14 H7 isolates were subjected to molecular characterization (Table 1 and Table 2). The molecular characteristics of whole-genome sequences were compared with those of reference sequences, including that of A/Italy/3/2013/H7N7, A/Jiangsu/1/2018/H7N4, and A/Shanghai/1/2013/H7N9. In contrast to the polybasic PKRRERR/GLF sequence of A/Jiangsu/1/2018/H7N4, most HA genes of H7 isolates encoded PELPKGR/GLF sequences at the cleavage site, which implies the low pathogenic status of the viruses. However, the H7N7 viruses isolated from Korea during 2018–19 lacked a well-known essential molecular marker linked with mammalian adaptation, such as Q226L in HA, similar to that of A/Jiangsu/1/2018/H7N4 and A/Shanghai/1/2013/H7N9. Furthermore, almost all of them lacked S138A mutation, known to be responsible for increased binding to mammalian receptors, and also present in the HA segment of A/Jiangsu/1/2018/H7N4. The identified mammalian adaptive markers, such as E627K and D701N in PB2, as well as specific amino acid changes at residue 66 in PB1-F2, residue 15 in M1, and residue 31 in M2, were not discovered in any of the H7 viruses. The P42S mutation in the NS1 protein was found in the majority of H7 isolates, which has been demonstrated to improve viral pathogenicity in mice.

### 3.3. Hypothesis for Reassortment Event in Each Gene Segment

**Figure 1:**
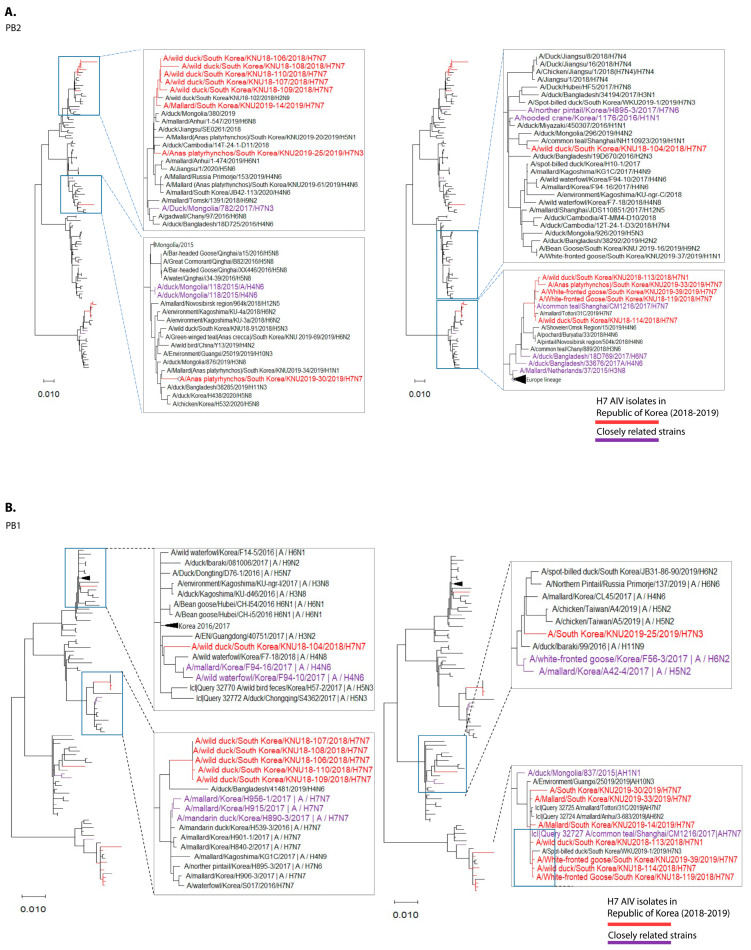

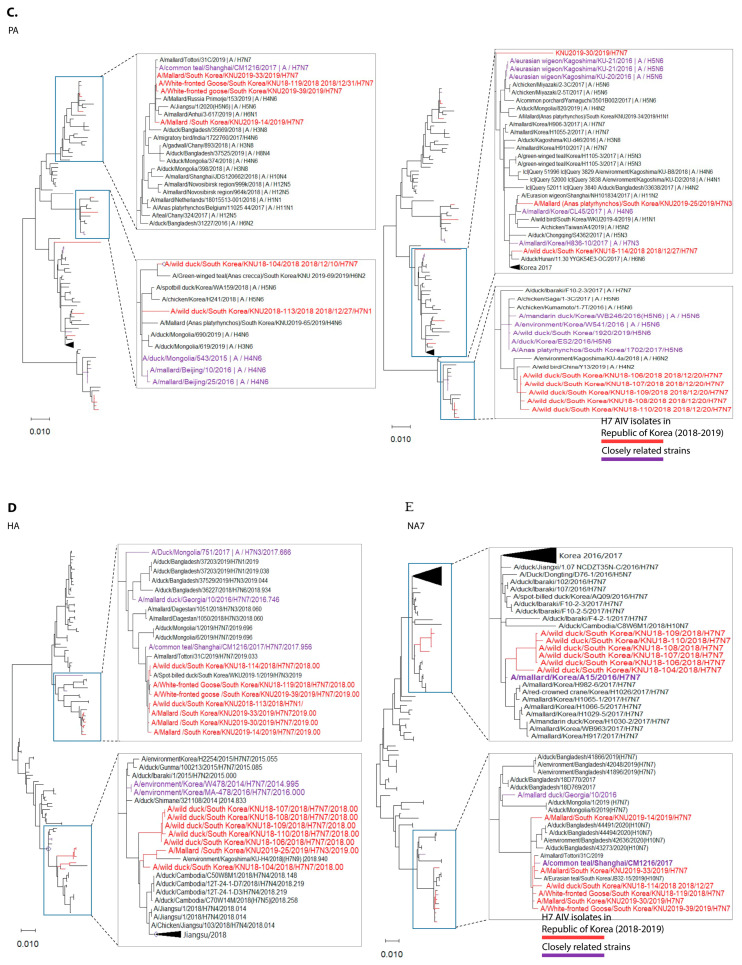

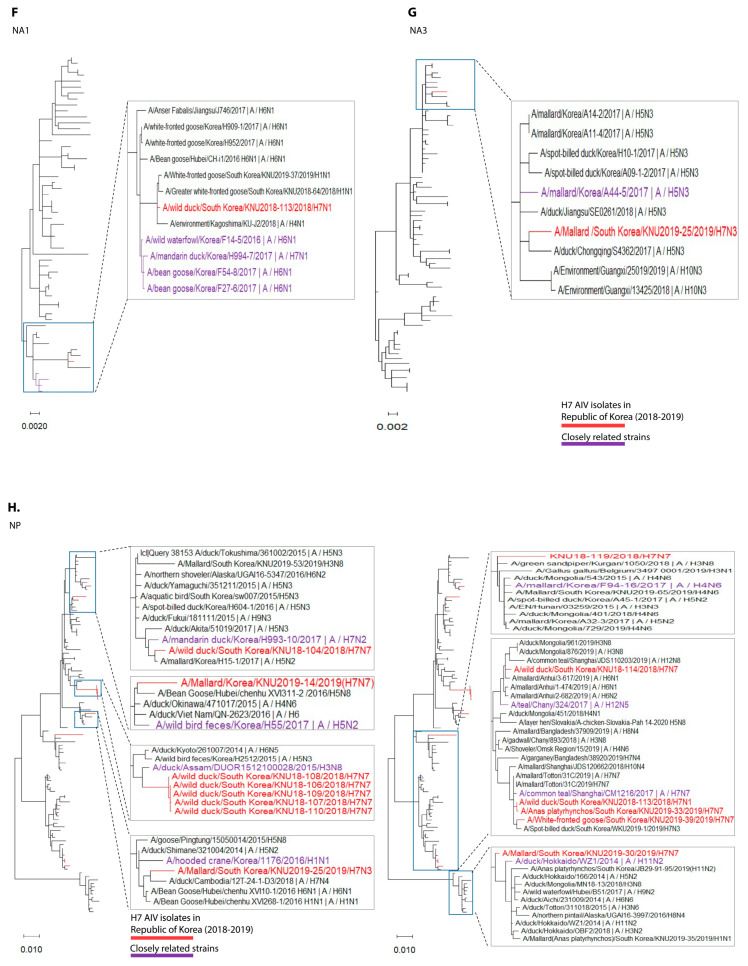

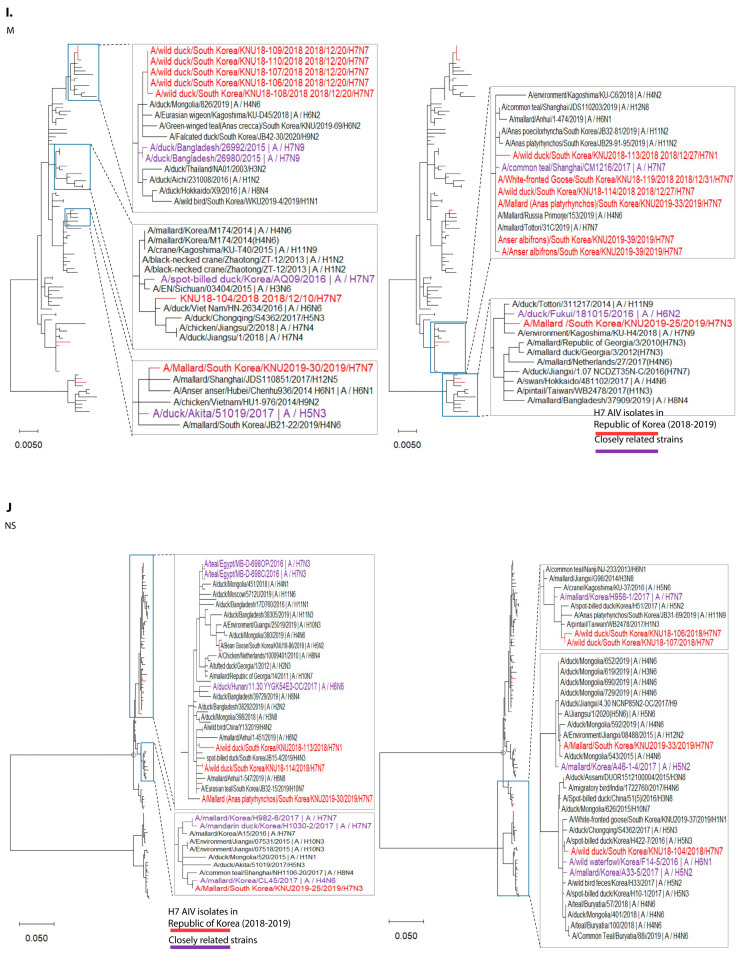
Maximum-likelihood phylogenetic trees of eight gene segments. Taxa are colored according to Korea H7 virus 2018/2019 isolates (red) and closely related strains (originated before 2018), based on highest nucleotide similarities to the 2018-2019 Korea H7 viruses (purple). (**A**) polymerase basic 2 (PB2); (**B**) polymerase basic 1 (PB1); (**C**) polymerase acidic (PA); (**D**) haemagglutinin (HA); (**E**) neuraminidase (NA7); (**F**) neuraminidase (NA1); (**G**) neuraminidase (NA3); (**H**) nucleoprotein (NP); (**I**) matrix (M); (**J**) non-structural (NS).

The maximum-likelihood technique implemented in MEGA v.6.017 was used to phylogenetically assess all eight segments of the H7 isolates, as well as accessible sequences downloaded from the NCBI Influenza Virus Resource and the Global Initiative on Sharing All Influenza Data (GISAID; http://www.gisaid.org) on 31 May 2021 (Figure 1).

**Figure 2:**
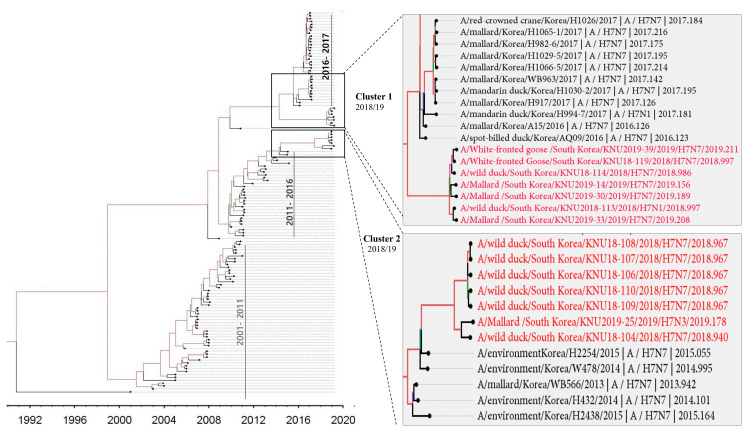
The SRD06 partitioned-substitution model, an uncorrelated lognormal relaxed clock, and a Bayesian skyline coalescent model in BEAST v1.10.4 were used to estimate the time-scaled maximum clade credibility (MCC) phylogeny for all HA sequences isolated between 1992–2019.

Through further evolutionary reassortment tracking analysis of our isolates (Figure 2), and on the basis of the available evidence, we proposed all H7 isolates from the Republic of Korea during 2018–2019 to be distinguishable into two clusters.

In cluster 1, involving isolates KNU106/2018, KNU107/2018, KNU108/2018, KNU110/2018, KNU109/2018, KNU104/2018, and KNU25/2019, NA, and HA gene reassortment prevailed in the H7N7 isolates from the Republic of Korea in 2016–2017. The highest sequence similarities of these isolates (~98%) with A/duck/Assam/DUOR1512100028/2015 (A/H3N8) predict inheritance of the NP gene, and that of the MP gene through ducks from Bangladesh (A/duck/Bangladesh/26980/2015 (A/H7N9)) and Fukui, Japan. The NS gene from A/teal/Egypt/2016 got reassorted into a duck in Hunan with H6N6, and further transferred to our isolates of category 1. The PB2/PA were predicted to be transmitted from isolates in Mongolia with H7N3 in 2017. All the isolates had Eurasian lineage.

**Figure 3:**
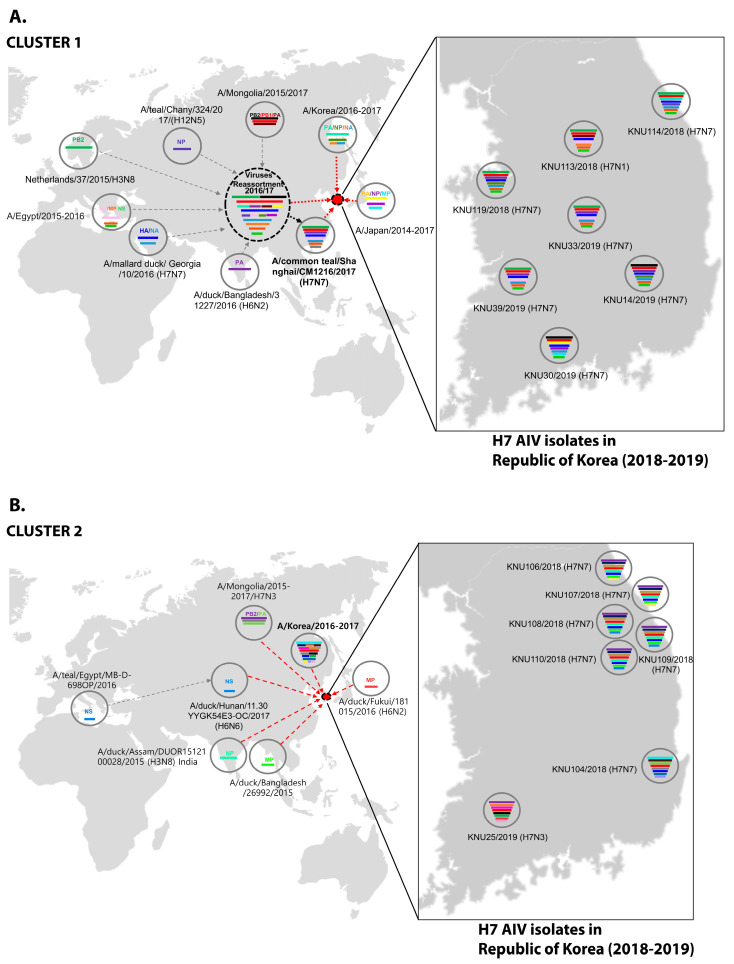
Original reassortment events of the novel avian influenza H7 isolates isolated during 2018–2019 in the Republic of Korea. (**A**) Cluster 1 represents possible reassortment events associated with KNU106/2018, KNU107/2018, KNU108/2018, KNU110/2018, KNU109/2018, KNU104/2018, and KNU25/2019. (**B**) Cluster 2 involves possible reassortment events associated with most of our isolates from 2019 and a few from 2018, such as KNU114, KNU113, and KNU119.

In cluster 2, involving most of our isolates from 2019 and a few from 2018, such as KNU114, KNU113, and KNU119, most of the segments were inherited from the reassorted viruses of 2017, the Korean isolates of 2016 and 2017, and from isolates in Japan from 2014–2016. The 2017 reassorted viruses possessed PB2 from Netherlands/2015/H3N8, NP/MP from A/teal/Chany/2017/H12N5-like viruses, NP/MP and NS from AIVs isolated from Egypt/2015-2016, HA/NA from A/mallard duck/Georgia/2016/H7N7, and PB2/PB1/PA from Mongolia/2015/2017. Detailed information on the evolutionary reassortment is presented in Figure 3.

## 4. Discussion

The major force guiding the evolution of AIVs is the adjustment with new hosts or the escape from the pre-existing host community [31]. This process of evolution is assisted by nucleotide substitutions involving amino acid mutations and reassortments [32]. In this study, we genetically characterized 14 H7 LP IAVs isolated from wild bird habitats in the Republic of Korea from 2018 to 2019. LPAIVs produce minor respiratory syndromes in chicken, as well as reduced feed intake and egg production, resulting in significant financial losses. The H7 subtype of AIVs is reported to infect a broad range of species, including wild birds, poultry, pigs, seals, and humans [33,34,35,36]. There have been instances of reassortment of H7 LP AIVs to HP AIVs. Ten outbreaks, caused by HP AIV, are known to have been preceded by circulation of a predecessor LP AIV in poultry [7]. Therefore, acute surveillance is necessary to prevent large scale economic losses.

We identified 26 changes in the human and similar avian H7 viruses’ genomes that could be involved in human adaptation or increased viral replication. Mutation in the HA segment may produce structural changes in the protein, affecting receptor binding affinity. All of the H7 viruses tested lacked Q226L and S138A [26] in HA, similar to that of A/Jiangsu/1/2018/H7N4 and A/Shanghai/1/2013/H7N9, known to be responsible for increased binding to mammalian receptors. This confirms their avian receptor binding specificity. Another important mutation, E627K [17] in the PB2 segment of human H7N4 virus, known for mammalian adaptation, was also absent in all of the isolates, further confirming their avian adaptivity. Apart from most of the mutations signifying avian host specificity, the P42S mutation in the NS1 protein was found in the majority of H7 isolates, which has been demonstrated to improve viral pathogenicity in mice, speculating further concerns for the mammalian adaption of these viruses in the future.

From our analysis, it is predicted that in 2018–2019, two different subgroups of H7 viruses might have been introduced into the Republic of Korea via different migratory bird populations through fecal contamination. The locations of circumpolar arctic and subarctic breeding grounds of migrating birds flying over numerous flyways in Eurasia overlap, with some sharing common breeding areas [37]. Thus, surveillance and monitoring of H7 subtype AIVs in wild birds are essential for avoiding large-scale morbidities.

On the basis of available resources, we proposed an evolutionary mechanism that could have led to the generation of novel H7 LPIAVs. The first phase of reassortment might have occurred among the cluster 1 viruses and other LP AIVs circulating in the Eurasian migratory bird pathway from 2015 to 2017. In the second phase, all the accumulated reassortments in the H7 viruses isolated between 2015–2016 from the Republic of Korea [14], together with other H7 viruses from other parts of Asia, such as Bangladesh, India, and Japan, may have led to the cluster 1 reassorted H7 isolates of 2018–2019. However, considering the HPAI outbreak that was caused by A/chicken/Italy/16vir1873/2016 (H7N7) in Italy in 2016, the unusually high prevalence of H7 viruses belonging to cluster I in wild bird habitats during the winter of 2018–2019 raised concerns of further outbreaks in poultry by this viral lineage.

In our proposed cluster 2 H7 reassorted viruses, the reassortments may have occurred through multiple phases. From phylodynamic analysis, it seemed that some H7 viruses of 2018–2019 had reemerged from the circulating viruses of 2011–2015. The PB2 segment from Netherlands/2015/H3N8, HA/NA from A/mallard/Georgia/2016/H7N7, PA from Bangladesh, and NP/MP from A/teal/Chany/H12N5-like viruses might have reassorted initially, and led to the evolution of a group of reassorted H7 viruses in 2017, which, further with segments from Korean isolates of 2016–2017 and isolates from Japan, might have led to the cluster 2 of H7 reassorted viruses of 2018–2019 in Korea. Thus, novel H7 reassortant viruses could have undergone further genetic reassortment with other LP IAVs, and spread across migratory bird species in these breeding sites prior to their subsequent dissemination during southern migration in 2016.

## 5. Conclusions

In summary, the study provided valuable information about the geographical distribution, representation, genetic diversity, and possible evolutionary pathways of novel H7 reassortants in wild bird habitats of the Republic of Korea during 2018–2019. H7 viruses in both the clusters with considerable genetic diversity were detected at an unusually high frequency in the winter of 2018–2019. The efficient subclinical replication and shedding of viruses by chickens might facilitate their silent spread among poultry. Phylogenetic and nucleotide sequence analyses of these isolates from the Republic of Korea revealed their distribution into two distinct clusters, with mutations that have been previously reported to be related to mammalian adaptation and changes in the virulence of AIVs. Thus, the recent appearance of novel H7 reassortants in migratory bird habitats highlighted the need for continued influenza surveillance in both wild birds and poultry in the Republic of Korea to prevent future pandemics, as well as huge economic losses.

## Figures and Tables

**Table 1:** Summary of data obtained from the mutational analysis of PB2, PB1, PA, and HA segments from the H7 isolates sampled during 2018–2019 in the Republic of Korea.

Virus	Sub-Type	PB2			PB1		PB1-F2	PA				HA		
		L89V	E627K	D701N	L13P	R207K	N66S	H266R	F277S	K356R	S409N	Cleavage Site	S138A	Q226L
		Markers of Mammalian Adaptation [17]		Increased Virulence in Mice [18]	Increased Polymerase Activity, Increased Virulence [19,20]		Increased virulence in Mammals [21]	Increased Polymerase Activity, Increased Virulence [22]	Increased Virulence, Mammalian Adaptation [23]	Increased Polymerase Activity, Increased Virulence in Mammals and Birds [24]	Enhanced Transmission, Marker of Mammalian Adaptation [25]		Increased Virus Binding to Human-Type Receptors [26]	
**A/Italy/3/2013**	H7N7	V	E	D	P	K	N	R	S	K	S	PELPKGR↓GLF	A	Q
**A/Jiangsu/1/2018**	H7N4	V	K	D	P	K	N	R	S	K	S	PKRRERR↓GLF	A	Q
**A/Shanghai/1/2013**	H7N9	V	K	D	P	K	N	R	S	R	N	PELPKGR↓GLF	S	Q
**KNU2018-104**	H7N7	V	E	D	P	K	N	R	S	K	S	PELPKGR↓GLF	A	Q
**KNU2018-106**	H7N7	V	E	D	P	K	N	R	S	K	S	PELPKGR↓GLF	A	Q
**KNU2018-107**	H7N7	V	E	D	P	K	N	R	S	K	S	PELPKGR↓GLF	A	Q
**KNU2018-108**	H7N7	V	E	D	P	K	N	R	S	K	S	PELPKGR↓GLF	A	Q
**KNU2018-109**	H7N7	V	E	D	P	K	N	R	S	K	S	PELPKGR↓GLF	A	Q
**KNU2018-110**	H7N7	V	E	D	P	K	N	R	S	K	S	PELPKGR↓GLF	A	Q
**KNU2018-113**	H7N1	V	E	D	P	K	S	R	S	K	S	PELPKGR↓GLF	A	Q
**KNU2018-114**	H7N7	V	E	D	P	K	S	R	S	K	S	PELPKGR↓GLF	A	Q
**KNU2018-119**	H7N7	V	E	D	P	K	S	R	S	K	S	PELPKGR↓GLF	A	Q
**KNU2019-14**	H7N7	V	E	D	P	K	S	R	S	K	S	PELPKGR↓GLF	A	Q
**KNU2019-33**	H7N7	V	E	D	P	K	S	R	S	K	S	PELPKGR↓GLF	A	Q
**KNU2019-39**	H7N7	V	E	D	P	K	S	R	S	K	S	PELPKGR↓GLF	A	Q
**KNU2019-25**	H7N3	V	E	D	P	K	N	R	F	K	S	PELPKGR↓GLF	A	Q
**KNU2019-30**	H7N7	V	E	D	P	K	S	R	S	K	S	PEPPKGR↓GLF	A	Q

**Table 2:** Summary of data obtained from the mutational analysis of NP, NA, M1, M2, and NS1 segments from the H7 isolates sampled during 2018–2019 in the Republic of Korea.

Virus	Sub-Type	NP			NA			M1			M2			NS1
		Virulence												
		V41I	A184K	D210E	M26I	I107V	R294K	V15I	N30D	T215A	I28V	S31N	L55F	P/A42S
		Enhanced RNP Activity [18]	Increased Virulence [27]	Enhanced RNP Activity [18]	Increased Virulence in Mammals		Resistant to Oseltamivir & Zanamivir Resistent [28]			Increased Virulence in Mice [29]	Mammalian Adaptation [30]			Increased Virulence in Mice
**A/Italy/3/2013**	H7N7	I	K	E	I	I	R	V	D	A	I	S	L	S
**A/Jiangsu/1/2018**	H7N4	I	K	E	L	I	R	V	D	A	I	S	L	S
**A/Shanghai/1/2013**	H7N9	I	K	E	I	V	K	I	D	A	V	N	F	S
**KNU2018-104**	H7N7	I	K	E	I	V	R	V	D	A	V	S	L	A
**KNU2018-106**	H7N7	I	K	E	I	V	R	V	D	A	I	S	L	S
**KNU2018-107**	H7N7	I	K	E	I	V	R	V	D	A	I	S	L	S
**KNU2018-108**	H7N7	I	K	E	I	V	R	V	D	A	I	S	L	S
**KNU2018-109**	H7N7	I	K	E	I	V	R	V	D	A	I	S	L	S
**KNU2018-110**	H7N7	I	K	E	I	V	R	V	D	A	I	S	L	S
**KNU2018-113**	H7N1	I	K	E	I	V	R	V	D	A	I	S	L	S
**KNU2018-114**	H7N7	I	K	E	I	V	R	V	D	A	I	S	L	S
**KNU2018-119**	H7N7	I	K	E	I	V	R	V	D	A	I	S	L	S
**KNU2019-14**	H7N7	I	K	E	I	V	R	V	D	A	I	S	L	S
**KNU2019-33**	H7N7	I	K	E	I	V	R	V	D	A	I	S	L	A
**KNU2019-39**	H7N7	I	K	E	I	V	R	V	D	A	I	S	L	S
**KNU2019-25**	H7N3	I	K	E	I	V	R	V	D	A	I	S	L	S
**KNU2019-30**	H7N7	I	K	E	I	V	R	V	D	A	I	S	L	S

## Data Availability

Data supporting reported results may be provided on reasonable request to the corresponding author.

## Supplementary Materials

The following are available online at https://www.mdpi.com/article/10.3390/v13112260/s1, Tables S1–S13: Individual gene segments of H7 AIV isolates from Korea during 2018–2019 with the highest nucleotide identity with respective strains obtained through nucleotide sequence blast in the influenza isolates database (GISAID). Tables S1–S13 represent the specific information about the H7 AIV isolates from Republic of Korea during 2018–2019.
