# Supplementary material for: Molecular Analysis of the Avian H7 Influenza Viruses Circulating in South Korea during 2018–2019: Evolutionary Significance and Associated Zoonotic Threats
Source: Viruses. 2021 Nov 11;13(11):2260. doi: 10.3390/v13112260 (PMC8623559; doi:10.3390/v13112260)
Supplement: Supplementary file 1 [file viruses-13-02260-s001.zip › viruses-1435497-supplementary.pdf]

*Article*

# Molecular analysis of the avian H7 influenza viruses circulating in South Korea during 2018–2019: Evolutionary significance and associated zoonotic threats

**Bao Tuan Duong [1†], Jyotiranjan Bal [1†], Haan Woo Sung [2*], Seon-Ju Yeo [3*] and Hyun Park [1*]**

## SUPPLEMENTARY TABLES

## Table Legends:

Tables S1-S13. Individual gene segments of H7 AIV isolates from Republic of Korea during 2018-2019 with the highest nucleotide identity with respective strains obtained through nucleotide sequence blast in the influenza isolates database (GISAID). S1-S13 represent the specific information about the H7 AIV isolates from Republic of Korea during 2018-2019

## Table S1

# KNU2019-14 (H7N7)

| Gene segment | Genebank ID | Reference strain | Country | Percent identity |
|---|---|---|---|---|
| PB2 | MW391557 | A/duck/Jiangsu/SE0261/2018(H5N3) | People's Republic of China | 99.12 |
| PB1 | MW391558 | A/common teal/Shanghai/CM1216/2017(H7N7) | People's Republic of China | 99.64 |
| PA | MW391559 | A/common teal/Shanghai/CM1216/2017(H7N7) | People's Republic of China | 99.44 |
| HA | MW391560 | A/common teal/Shanghai/CM1216/2017(H7N7) | People's Republic of China | 99.51 |
| NP | MW391561 | A/duck/Viet Nam/QN-2623/2016(H6) | Vietnam | 98.80 |
| NA | MW391562 | A/mallard duck/Georgia/10/2016(H7N7) | Georgia | 97.88 |
| M1/M2 | MW391563 | A/Duck/Mongolia/652/2017(H7N3) | Mongolia | 99.12 |
| NS1/NS2 | MW391564 | A/wild goose/dongting lake/121/2018(H6N2)) | People's Republic of China | 99.53 |

## Table S2

# KNU2019-33 (H7N7)

| Gene segment | Genebank ID | Reference strain | Country | Percent identity |
|---|---|---|---|---|
| PB2 | MW391497 | A/common teal/Shanghai/CM1216/2017(H7N7) | People's Republic of China | 99.37 |
| PB1 | MW391498 | A/common teal/Shanghai/CM1216/2017(H7N7) | People's Republic of China | 99.82 |
| PA | MW391499 | A/common teal/Shanghai/CM1216/2017(H7N7) | People's Republic of China | 99.77 |
| HA | MW391500 | A/common teal/Shanghai/CM1216/2017(H7N7) | People's Republic of China | 99.63 |
| NP | MW391501 | A/common teal/Shanghai/CM1216/2017(H7N7) | People's Republic of China | 99.67 |
| NA | MW391502 | A/common teal/Shanghai/CM1216/2017(H7N7) | People's Republic of China | 99.63 |
| M1/M2 | MW391503 | A/common teal/Shanghai/CM1216/2017(H7N7) | People's Republic of China | 99.90 |
| NS1/NS2 | MW391504 | A/duck/Jiangxi/4.30_NCNP85N2-OC/2017(mixed) | People's Republic of China | 99.77 |

## Table S3

# KNU2019-33 (H7N7)

| Gene segment | Genebank ID | Reference strain | Country | Percent identity |
|---|---|---|---|---|
| PB2 | MW391497 | A/common teal/Shanghai/CM1216/2017(H7N7) | People's Republic of China | 99.37 |
| PB1 | MW391498 | A/common teal/Shanghai/CM1216/2017(H7N7) | People's Republic of China | 99.82 |
| PA | MW391499 | A/common teal/Shanghai/CM1216/2017(H7N7) | People's Republic of China | 99.77 |
| HA | MW391500 | A/common teal/Shanghai/CM1216/2017(H7N7) | People's Republic of China | 99.63 |
| NP | MW391501 | A/common teal/Shanghai/CM1216/2017(H7N7) | People's Republic of China | 99.67 |
| NA | MW391502 | A/common teal/Shanghai/CM1216/2017(H7N7) | People's Republic of China | 99.63 |
| M1/M2 | MW391503 | A/common teal/Shanghai/CM1216/2017(H7N7) | People's Republic of China | 99.90 |
| NS1/NS2 | MW391504 | A/duck/Jiangxi/4.30_NCNP85N2-OC/2017(mixed) | People's Republic of China | 99.77 |

# Table S4

## KNU2019-25 (H7N3)

| Gene segment | Genebank ID | Reference strain | Country | Percent identity |
|---|---|---|---|---|
| PB2 | MW391567 | A/duck/Jiangsu/SE0261/2018(H5N3) | People's Republic of China | 99.12 |
| PB1 | MW391568 | A/duck/Cambodia/C8W6M1/2018(H10N7) | Cambodia | 98.68 |
| PA | MW391569 | A/wild goose/dongting lake/121/2018(H6N2) | People's Republic of China | 98.91 |
| HA | MW391570 | A/Jiangsu/1/2018(H7N4) | People's Republic of China | 98.22 |
| NP | MW391571 | A/duck/Cambodia/12T-24-1-D17/2018(H7N4) | Cambodia | 98.88 |
| NA | MW391572 | A/pintail/Taiwan/WB2478/2017(H1N3) | Taiwan | 99.23 |
| M1/M2 | MW391573 | A/duck/Hokkaido/OBF2/2018(H3N2) | Japan | 99.49 |
| NS1/NS2 | MW391574 | A/duck/Mongolia/520/2015(H1N1) | Mongolia | 99.43 |

**Table S5**

| KNU2019-30 (H7N7) | | | | |
|---|---|---|---|---|
| Gene segment | Genebank ID | Reference strain | Country | Percent identity |
| PB2 | MW404521 | A/duck/Mongolia/30/2015 (A/H3N8) segment 1 (PB2) | Mongolia | 97 |
| PB1 | MW404522 | A/common teal/Shanghai/CM1216/2017(H7N7) | People's Republic of China | 99 |
| PA | MW404523 | A/eurasian wigeon/Kagoshima/KU-20/2016 (A/H5N6) segment 3 (PA)) | Japan | 97 |
| HA | MW404524 | A/common teal/Shanghai/CM1216/2017(H7N7)) | People's Republic of China | 99.63 |
| NP | MW404525 | A/duck/Hokkaido/WZ1/2014(H11N2) | Japan | 99.27 |
| NA | MW404526 | A/common teal/Shanghai/CM1216/2017(H7N7) | People's Republic of China | 99.63 |
| M1/M2 | MW404527 | A/duck/Akita/51019/2017(H5N3) | Japan | 99.69 |
| NS1/NS2 | MW404528 | A/wild goose/dongting lake/121/2018(H6N2) | People's Republic of China | 99.19 |

## Table S6

# KNU2018-104 (H7N7)

| Gene segment | Genebank ID | Reference strain | Country | Percent identity |
|---|---|---|---|---|
| PB2 | MN483242 | A/duck/Miyazaki/450307/2016 (A/H1N1) segment 1 (PB2) | Mongolia | 98 |
| PB1 | MN483243 | A/wild bird feces/Korea/H189-1/2016 (A/H5N3) segment 2 (PB1) | Republic of Korea | 99 |
| PA | MN483244 | A/duck/Mongolia/543/2015 (A/H4N6) segment 3 (PA) | Mongolia | 99 |
| HA | MN483245 | A/duck/Ibaraki/1/2015 (A/H7N2) segment 4 (HA) | Japan | 98 |
| NP | MN483246 | A/mallard/Korea/H15-1/2017 (A/H5N2) segment 5 (NP) | Republic of Korea | 99 |
| NA | MN483247 | A/mallard/Korea/A15/2016 (A/H7N7) segment 6 (NA) | Republic of Korea | 98 |
| M1/M2 | MN483248 | A/duck/Viet Nam/HN-2634/2016 (A/H6N6) segment 7 (MP) | Vietnam | 99 |
| NS1/NS2 | MN483249 | A/spot-billed duck/Korea/H422-7/2016 (A/H5N3) segment 8 (NS) | Republic of Korea | 99 |

## Table S7

# KNU2018-106 (H7N7)

| Gene segment | Genebank ID | Reference strain | Country | Percent identity |
|---|---|---|---|---|
| PB2 | MN483208 | A/duck/Mongolia/769/2015 (A/H4N6) segment 1 (PB2) | Mongolia | 98 |
| PB1 | MN483209 | A/mallard/Korea/WA245/2017 (A/H7N7) segment 2 (PB1) | Republic of Korea | 98 |
| PA | MN483210 | A/duck/Ibaraki/F10-2-5/2017 (A/H7N7) segment 3 (PA) | Japan | 99 |
| HA | MN483211 | A/environment/Korea/MA-478/2016 (A/H7N7) segment 4 (HA) | Republic of Korea | 98 |
| NP | MN483212 | A/duck/Assam/DUOR1512100028/2015 (A/H3N8) segment 5 (NP) | India | 98 |
| NA | MN483213 | A/mallard/Korea/A15/2016 (A/H7N7) segment 6 (NA) | Republic of Korea | 98 |
| M1/M2 | MN483214 | A/duck/Bangladesh/26980/2015 (A/H7N9) segment 7 (MP) | Bangladesh | 99 |
| NS1/NS2 | MN483215 | A/spot-billed duck/Korea/H422-7/2016 (A/H5N3) segment 8 (NS) | Republic of Korea | 99 |

## Table S8

# KNU2018-107 (H7N7)

| Gene segment | Genebank ID | Reference strain | Country | Percent identity |
|---|---|---|---|---|
| PB2 | MN483250 | A/duck/Mongolia/769/2015 (A/H4N6) segment 1 (PB2) | Mongolia | 98 |
| PB1 | MN483251 | A/mallard/Korea/WA245/2017 (A/H7N7) segment 2 (PB1) | Republic of Korea | 98 |
| PA | MN483252 | A/duck/Ibaraki/F10-2-5/2017 (A/H7N7) segment 3 (PA) | Japan | 99 |
| HA | MN483253 | A/environment/Korea/MA-478/2016 (A/H7N7) segment 4 (HA) | Republic of Korea | 97 |
| NP | MN483254 | A/duck/Assam/DUOR1512100028/2015 (A/H3N8) segment 5 (NP) | India | 98 |
| NA | MN483255 | A/mallard/Korea/A15/2016 (A/H7N7) segment 6 (NA) | Republic of Korea | 98 |
| M1/M2 | MN483256 | A/duck/Bangladesh/26980/2015 (A/H7N9) segment 7 (MP) | Bangladesh | 99 |
| NS1/NS2 | MN483257 | A/wild bird feces/Korea/H49/2017 (A/H5N2) segment 8 (NS) | Republic of Korea | 99 |

# Table S9

## KNU2018-108 (H7N7)

| Gene segment | Genebank ID | Reference strain | Country | Percent identity |
|---|---|---|---|---|
| PB2 | MN483215 | A/duck/Mongolia/769/2015 (A/H4N6) segment 1 (PB2) | Mongolia | 96 |
| PB1 | MN483216 | A/mallard/Korea/WA245/2017 (A/H7N7) segment 2 (PB1) | Republic of Korea | 98 |
| PA | MN602497 | A/duck/Ibaraki/F10-2-5/2017 (A/H7N7) segment 3 (PA) | Japan | 99 |
| HA | MN483217 | A/environment/Korea/MA-478/2016 (A/H7N7) segment 4 (HA) | Republic of Korea | 97 |
| NP | MN483218 | A/duck/Assam/DUOR1512100028/2015 (A/H3N8) segment 5 (NP) | India | 98 |
| NA | MN483219 | A/mallard/Korea/A15/2016 (A/H7N7) segment 6 (NA) | Republic of Korea | 98 |
| M1/M2 | MN483220 | A/duck/Bangladesh/26980/2015 (A/H7N9) segment 7 (MP) | Bangladesh | 99 |
| NS1/NS2 | MN602498 | A/teal/Egypt/MB-D-698C/2016 (A/H7N3) segment 8 (NS) | Egypt | 99 |

## Table S10

# KNU2018-109 (H7N7)

| Gene segment | Genebank ID | Reference strain | Country | Percent identity |
|---|---|---|---|---|
| PB2 | MN483221 | A/duck/Mongolia/769/2015 (A/H4N6) segment 1 (PB2) | Mongolia | 98 |
| PB1 | MN483222 | A/mallard/Korea/WA245/2017 (A/H7N7) segment 2 (PB1) | Republic of Korea | 98 |
| PA | MN602499 | A/duck/Ibaraki/F10-2-5/2017 (A/H7N7) segment 3 (PA) | Japan | 99 |
| HA | MN483223 | A/environment/Korea/MA-478/2016 (A/H7N7) segment 4 (HA) | Republic of Korea | 98 |
| NP | MN602500 | A/duck/Assam/DUOR1512100028/2015 (A/H3N8) segment 5 (NP) | India | 98 |
| NA | MN483224 | A/mallard/Korea/A15/2016 (A/H7N7) segment 6 (NA) | Republic of Korea | 98 |
| M1/M2 | MN483220 | A/duck/Bangladesh/26980/2015 (A/H7N9) segment 7 (MP) | Bangladesh | 99 |
| NS1/NS2 | MN602501 | A/teal/Egypt/MB-D-698C/2016 (A/H7N3) segment 8 (NS) | Egypt | 99 |

## Table S11

# KNU2018-110 (H7N7)

| Gene segment | Genebank ID | Reference strain | Country | Percent identity |
|---|---|---|---|---|
| PB2 | MN483226 | A/duck/Mongolia/769/2015 (A/H4N6) segment 1 (PB2) | Mongolia | 98 |
| PB1 | MN483227 | A/mallard/Korea/WA245/2017 (A/H7N7) segment 2 (PB1) | Republic of Korea | 98 |
| PA | MN602502 | A/duck/Ibaraki/F10-2-5/2017 (A/H7N7) segment 3 (PA) | Japan | 99 |
| HA | MN483228 | A/environment/Korea/MA-478/2016 (A/H7N7) segment 4 (HA) | Republic of Korea | 97 |
| NP | MN483229 | A/duck/Assam/DUOR1512100028/2015 (A/H3N8) segment 5 (NP) | India | 98 |
| NA | MN602503 | A/mallard/Korea/A15/2016 (A/H7N7) segment 6 (NA) | Republic of Korea | 98 |
| M1/M2 | MN483230 | A/duck/Bangladesh/26980/2015 (A/H7N9) segment 7 (MP) | Bangladesh | 99 |
| NS1/NS2 | MN602504 | A/teal/Egypt/MB-D-698C/2016 (A/H7N3) segment 8 (NS) | Egypt | 99 |

## Table S12

# KNU2018-114 (H7N7)

| Gene segment | Genebank ID | Reference strain | Country | Percent identity |
|---|---|---|---|---|
| PB2 | MN483231 | A/common teal/Shanghai/CM1216/2017 (A/H7N7) segment 1 (PB2) | People's Republic of China | 99 |
| PB1 | MN602505 | A/common teal/Shanghai/CM1216/2017 (A/H7N7) segment 2 (PB1) | People's Republic of China | 99 |
| PA | MN602506 | A/mallard/Korea/H836-10/2017 (A/H7N3) segment 3 (PA) | Japan | 99 |
| HA | MN483232 | A/common teal/Shanghai/CM1216/2017 (A/H7N7) segment 4 (HA) | People's Republic of China | 99 |
| NP | MN483233 | A/teal/Chany/324/2017 (A/H12N5) segment 5 (NP) | Russia | 99 |
| NA | MN483234 | A/duck/Bangladesh/18D769/2017 (A/H6N7) segment 6 (NA) | Bangladesh | 98 |
| M1/M2 | MN483235 | A/common teal/Shanghai/CM1216/2017 (A/H7N7) segment 7 (MP) | People's Republic of China | 99 |
| NS1/NS2 | MN602507 | A/teal/Egypt/MB-D-698C/2016 (A/H7N3) segment 8 (NS) | Egypt | 99 |

## Table S13

# KNU2018-119 (H7N7)

| Gene segment | Genebank ID | Reference strain | Country | Percent identity |
|---|---|---|---|---|
| PB2 | MN483236 | A/duck/Bangladesh/18D770/2017 (A/H6N7) segment 1 (PB2) | Bangladesh | 98 |
| PB1 | MN602508 | A/common teal/Shanghai/CM1216/2017 (A/H7N7) segment 2 (PB1) | People's Republic of China | 99 |
| PA | MN602509 | A/common teal/Shanghai/CM1216/2017 (A/H7N7) segment 3 (PA) | People's Republic of China | 99 |
| HA | MN483237 | A/common teal/Shanghai/CM1216/2017 (A/H7N7) segment 4 (HA) | People's Republic of China | 99 |
| NP | MN483238 | A/mallard/Korea/F94-16/2017 (A/H4N6) segment 5 (NP) | Republic of Korea | 97 |
| NA | MN483239 | A/duck/Bangladesh/18D769/2017 (A/H6N7) segment 6 (NA) | Bangladesh | 98 |
| M1/M2 | MN483240 | A/common teal/Shanghai/CM1216/2017 (A/H7N7) segment 7 (MP) | People's Republic of China | 99 |
| NS1/NS2 | MN483241 | A/teal/Egypt/MB-D-698C/2016 (A/H7N3) segment 8 (NS) | Egypt | 99 |
